# Acceptance and barriers to cervical cancer screening among mothers in a state-capital city: a descriptive cross-sectional study

**DOI:** 10.3332/ecancer.2025.1916

**Published:** 2025-05-29

**Authors:** Afusat O Oduwoye, Elisha O Olabisi, Elizabeth F Ojo, Taiwo O Dosumu, Michael O Owoeye, Adelani Tijani, Deborah T Esan

**Affiliations:** 1 Community Health Department, Osun State University Teaching Hospital, Osogbo 230001, Nigeria; 2 Faculty of Nursing Sciences, Ladoke Akintola University of Technology, Ogbomoso 210214, Nigeria; 3 Department of Nursing Science, Afe Babalola University, Ado-Ekiti 360231, Nigeria; 4 Faculty of Nursing Sciences, Bowen University, Iwo 232101, Nigeria; 5 Sociology Programme, Bowen University, Iwo 232101, Nigeria; 6 Department of Nursing, Federal University, Oye-Ekiti 371101, Nigeria

**Keywords:** acceptance, cervical cancer, Nigeria, Osogbo, screening, susceptibility

## Abstract

**Background:**

Cervical cancer is a reproductive malignancy that may be detected in its pre-invasive stage by regular cytological screening,

**Objective:**

This study assessed the acceptance of cervical cancer screening among mothers attending infant welfare clinics in hospitals in Osogbo, Osun State, Nigeria.

**Methods:**

A descriptive cross-sectional design was employed, with questionnaires administered to 355 respondents, selected using Fisher’s formula. A multistage sampling technique was used to ensure a representative sample. Data were analysed using descriptive and inferential statistical methods. Relationships between variables were tested using chi-square and Fisher’s exact tests at a 5% significance level.

**Result:**

Approximately half (46.9%) had adequate knowledge of cervical cancer and its screening. More than half (55.7%) had low susceptibility to cervical cancer. About two-thirds (61.6%) were willing to undergo cervical cancer screening while only a quarter (25.9%) had undergone the screening test. Barriers to cervical cancer screening include lack of awareness, fear, perceived immunity, cost, pain and embarrassment while motivators include integration with general health screenings and perceived necessity. Findings suggest there is a statistically significant association between mothers’ age, marital status, ethnicity, knowledge of cervical cancer, susceptibility to cervical cancer and cervical cancer screening acceptability.

**Conclusion:**

Acceptance of screening was high which was significantly influenced by their degree of knowledge and susceptibility to cervical cancer. However, screening acceptance was not consistent with their low uptake.

**Implications for practice:**

There is a need for continuous education and policies to minimise costs and ensure accessibility to the screening test to promote and improve its uptake thus reducing morbidities and mortalities associated with the disease.

## Introduction

Cervical cancer continues to be a significant public health concern, particularly in developing nations such as Nigeria, where it is the most prevalent reproductive malignancy [1]. The disease progresses silently until it reaches an advanced stage, making early detection through regular screenings critical [2]. Cervical cancer can be detected in its pre-invasive phase through various screening methods including cytological approaches, such as the Papanicolaou (Pap) smear and liquid-based cytology, which has historically formed the basis of screening programs [2–4]. Today, human papillomavirus (HPV) DNA testing is prioritised by the World Health Organisation (WHO) as the most sensitive method for primary screening as large-scale clinical trials have consistently demonstrated the high sensitivity of HPV DNA testing in detecting high-risk HPV infections that are more likely to progress to cervical cancer [3, 4]. In resource-limited settings, visual inspection with acetic acid (VIA) or Lugol’s iodine (VILI) is often used due to their low cost and simplicity [5]. Methods like HPV mRNA testing may be used as primary screening methods but the scope of investigation is still emerging. Collectively, these strategies enable early detection and treatment of precancerous lesions, significantly reducing the incidence of invasive cervical cancer [2, 4].

Cervical cancer most commonly affects women aged 30 to 45, but can affect women of all ages, with cases documented in women as young as 18 [1]. Regular cervical cancer screening, usually at 3-year intervals, has been highlighted in most scholarly evidence [6]. Hammer *et al* [7] suggest that women who have had three consecutive negative screenings within a decade may consider stopping screenings around the age of 65, provided they have no history of cervical cancer or pre-cancerous lesions. However, more recent evidence from the WHO recommends HPV DNA testing for cervical cancer prevention starting at age 30 (every 5–10 years) via screen-and-treat or screen-triage-treat. For women with HIV, screening begins earlier (age 25 years) at 3–5 years intervals, using screen-triage-treat [4].

While most developed countries have well-established cervical cancer screening programs, countries like Nigeria lack such organised efforts, which significantly impacts the early detection and treatment of the disease [1, 8]. The WHO [9] recommends cervical cancer screening, ideally between the ages of 35 and 40 due to the high risks among this demographic [10, 11]. Previous studies had shown varying levels of acceptance, and suboptimal uptake of cervical cancer screening among samples of the Nigerian population. An integrative review by Uchendu *et al* [12], revealed the knowledge of cervical cancer and the uptake of screening was poor among the general Nigeria population. Also, a study by Akintobi *et al* [13] in, South-Western Nigeria found out less than one-third have had cervical cancer screening done before, with barriers such as lack of knowledge on the screenings and low perceived susceptibility due to the absence of signs and symptoms of cervical cancer.

Furthermore, a study by Maitanmi *et al* [14] among female undergraduates in Southwestern Nigeria revealed that only approximately half were ready to undergo cervical cancer screening. Conversely, studies in South-Southern Nigeria [15, 16] revealed majority were willing to accept cervical cancer screening. However, considering the dearth of these findings in this resource setting, this study, aims to explore the acceptability of cervical cancer screening among mothers attending infant welfare clinics in hospitals in Osogbo, Osun State thus, informing public health policies and programs designed to reduce the incidence, sequalae and mortalities of cervical cancer in this region.

The study addressed the following research questions:

1. What is the level of cervical cancer knowledge among the mothers?
2. What is the level of acceptance of cervical cancer screening among the mothers?
3. Is there a significant relationship between socio-demographic factors and cervical cancer screening history among the mothers?

## Materials and method

### Study design and setting

This descriptive cross-sectional study was conducted from June to August 2022 in five hospitals in Osogbo, Nigeria: UNIOSUN Teaching Hospital (tertiary care), Asubiaro Specialist State Hospital (secondary care) and three primary healthcare centres (Akogun, Isale Agbara and Atelewo). These facilities were selected to represent different levels of healthcare provision in the region.

### Population and sampling

The study population comprised women aged 18 and above attending the selected facilities. We excluded women who were mentally incapacitated or unwilling to participate. The sample size for the study was calculated using Fisher’s formula (*n* = Z²pq/d²), considering a 95% confidence interval (*z* = 1.96; 95% confidence interval), a precision of 0.05 (*d* = 0.05) and a prevalence of cervical screening acceptability at 70% (*p* = 0.7) [14]. This yielded a sample size of 323. We increased this by 10% to account for potential non-responses, resulting in a final sample size of 355.

We employed a multistage sampling technique: Hospitals were stratified into tertiary, secondary and primary care levels. One tertiary, one secondary and three primary care facilities were randomly selected. The sample size was distributed proportionally based on the average monthly patient flow in each selected facility. Within each facility, participants were selected using systematic random sampling based on the clinic’s attendance register. The distribution of participants across facilities was as follows: UNIOSUN Teaching Hospital (120), Asubiaro Specialist State Hospital (95), Akogun Primary Health Care Centre (50), Isale Agbara Primary Health Care Centre (45) and Atelewo Primary Health Care Centre (45).

### Research instrument

A structured questionnaire, adapted from Al-Amro *et al* [8], was used for data collection. The questionnaire was translated into both English and Yoruba to accommodate respondents’ language preferences. It consisted of five sections: Section A gathered socio-demographic data; Section B assessed the participants’ knowledge of cervical cancer and screening; Section C evaluated their perceived susceptibility to cervical cancer; Section D focused on their acceptance of cervical cancer screening and Section E explored the factors influencing their acceptance of screening.

The content validity of the instrument was ensured through an assessment conducted by experts in obstetrics and gynaecology. To establish face validity, a subset of the target population, consisting of 20 women, evaluated the instrument to confirm its clarity and relevance. Additionally, the reliability of the instrument was tested through a pilot study involving 20 women who were not part of the main study. Test-retest reliability was measured over a 1-week interval, with Pearson’s Correlation Coefficients ranging from 0.75 to 0.80 for the knowledge, susceptibility and acceptance subscales, indicating a high level of consistency.

### Data collection

Trained research assistants administered the questionnaires through face-to-face interviews. This method was selected to ensure that participants fully understood the questions, allowing the interviewers to provide clarifications when necessary. It also aimed to maximise response rates and minimise the occurrence of missing data. The interviewers received training to maintain neutrality and avoid influencing the participants’ responses. To ensure confidentiality, the interviews were conducted in private areas within the healthcare facilities.

### Ethical consideration

Ethical approval was obtained from the Ethics and Research Committee of Osun State Ministry of Health (OSHREC/PRS/569T/262). Informed consent was obtained from all participants. The study adhered to the principles of the Declaration of Helsinki.

### Data analysis

Data analysis was performed using IBM SPSS version 25. Descriptive statistics, including frequencies, percentages, means and standard deviations, were calculated to summarise the data. For inferential statistics, chi-square tests were conducted to explore relationships between socio-demographic factors and cervical cancer screening history, while Fisher’s exact test was used for cells with contingency tables.

Knowledge about cervical cancer was assessed through 25 questions, with each correct answer earning one point. Adequate knowledge was defined as a score at or above the 50th percentile. Susceptibility to cervical cancer was evaluated using risk factor questions, scored 1 for ‘yes’ and 0 for ‘no,’ with low susceptibility defined as a score of 0 and high susceptibility for any score above 0. Acceptance of cervical cancer screening was analysed as a binary outcome, categorised as either willing or not willing to undergo screening. The results were presented in tables and charts, accompanied by appropriate statistical measures such as chi-square values and *p*-values, where relevant.

## Results

Out of the calculated sample size of 355, a total of 305 respondents fully completed the questionnaire, resulting in a response rate of 85.9%.

Table 1 presents the socio-demographic characteristics of the respondents. The majority were aged 28–37 years (46.2%), predominantly Christians (63.3%), of Yoruba ethnicity (90.2%) and married (83.3%). Most had tertiary education (76.4%), and a large proportion were employed (63.3%). More than half (55.7%) of respondents had low susceptibility (no risk factors but had been sexually active), while 44.3% had one or more risk factors with 19.3% being sexually active before age 18, 17.7% having been treated for chlamydia and 17.7% having given birth more than three times. Conversely, most reported ‘No’ to factors associated with increased susceptibility, including smoking cigarettes (99.0%), HIV infection (99.0%), use of immunosuppressant drugs (98.0%), family history of cervical cancer (97.0%), use of diethylstilbestrol (95.7%), long-term use of oral contraceptives (93.1%) and having a baby before age 18 (93.1%). On average, only 7.9% of respondents might be susceptible to cervical cancer.

Table 2 summarises respondents’ knowledge of cervical cancer and its screening. Over half of the respondents recognised vaginal bleeding (55.4%) and foul-smelling discharge (54.1%) as symptoms of cervical cancer, but 53.4% did not identify pain during sex as a symptom. The most known risk factors were multiple sexual partners (44.3%) and HPV infection (42%), though 63.2% were unaware of most risk factors associated with cervical cancer. Respondents displayed strong knowledge of prevention methods, with 67.5% aware that avoiding multiple sexual partners can prevent cervical cancer, followed by avoiding early sexual intercourse (64.5%), not smoking (63.9%), early screening (63%) and avoiding long-term contraceptive use (61.6%). Regarding treatment, 51.8% knew about surgery and 50.5% about chemotherapy, but 59.7% lacked knowledge about radiotherapy as a treatment option. Knowledge of screening intervals was limited, with 41.6% suggesting annual screening, 40.3% 3-year intervals and 24.3% 5-year intervals. Many respondents were unaware of the eligibility criteria for cervical cancer screening, with only 42.3% knowing it is for women aged 30 and above. Overall, (Table 1) less than half (46.9%) of respondents had adequate knowledge of cervical cancer and its screening, while 53.1% had inadequate knowledge.

Table 3 shows most respondents (61.6%) expressed interest in undergoing a pap smear or cervical cancer screening. Only 25.9% reported having had a screening test previously. Among those screened, most (79.7%) had done so once, 11.4% had been screened twice, with 63.3% undergoing screening every 3 years, 20.3% annually and 16.5% at random intervals. The types of screening varied, with 65.8% having visual inspection with acetic acid and 32.2% pap smears. The majority (70.8%) believed women of reproductive age would participate in screening, and 74.1% would recommend it to a female friend.

Table 4 presents the reasons for non-utilisation of cervical cancer screening services among respondents. ‘Agree’ responses include both ‘strongly agree’ and ‘agree,’ while ‘disagree’ responses combine ‘strongly disagree’ and ‘disagree.’ The majority (62.4%) were unaware of the screening services, 60.6% expressed fears about potential harm due to substandard equipment or errors by experts. and 58.4% believed they were not at risk for cervical cancer. Additionally, 48.2% cited fear of exposure to other diseases, 37.6% felt the procedure was painful, 33.2% considered it too expensive and 29.6% found it embarrassing. However, the primary reason cited for utilisation includes awareness and feeling of necessity (79.7%), followed by the screening being part of general health checks (73.4%) and the availability of free or subsidised services (68.4%). Moreover, significant associations were found between cervical cancer screening history and several socio-demographic variables of respondents: Age (*p* = 0.004), ethnicity (*p* = 0.021), marital status (*p* = 0.005), knowledge level (*p* = 0.049) and perceived susceptibility (*p* = 0.036). Specifically, older age groups, Igbos and widowed individuals were more likely to have been screened. Those with adequate knowledge and higher perceived susceptibility were also more likely to undergo screening. No significant associations were found for religion (*p* = 0.72), educational level (*p* = 0.91) and occupation (*p* = 0.597).

## Discussion

The study population, consisting of mainly young adults (28–37 years), is consistent with the female reproductive age cycle in line with those reported in other similar studies [17, 18]. A minority having early sexual initiation (before age 18), a history of chlamydia treatment or multiparity (more than three births) are at higher risk of cervical cancer warranting evidence-based preventive measures such as cervical cancer screening [2].

The study found that approximately half of the mothers had adequate knowledge of cervical cancer and screening, with recognition of some symptoms but poor awareness of risk factors. Knowledge of prevention methods was higher for behaviours such as avoiding multiple sexual partners, early sexual intercourse and smoking. However, screening knowledge was limited, with confusion surrounding eligibility and necessity, indicating the need for more comprehensive education on cervical cancer. Similarly, a study by Olubodun *et al* [19] in Lagos, Nigeria, found poor knowledge of cervical cancer, screening and HPV immunisation among women in urban slums. Akpo *et al* [20] also reported high ignorance of cervical cancer risk factors among female students in the Commonwealth of Dominica. This pattern of poor knowledge has persisted for over a decade, as demonstrated by Abotchie and Shokar [18] and Dursun *et al* [17], who highlighted suboptimal awareness of the HPV-cervical cancer link among populations in Ghana and Turkey, respectively, underscoring a significant knowledge gap.

In contrast, Toye *et al* [21], who conducted a similar study in Lagos, Southwestern Nigeria, found a higher level of knowledge about cervical cancer and its prevention among high school teachers in the Mushin Local Government Area. The discrepancy in knowledge levels between mothers attending infant welfare clinics in the current study and the high school teachers studied by Toye *et al* [21] may be attributed to several factors. Socioeconomic status likely plays a role, as higher education levels are often associated with better access to information and healthcare resources [22]. Additionally, targeted educational programs or interventions for high school teachers may have contributed to their superior knowledge of cervical cancer [23].

Findings indicate high acceptance of cervical cancer screening, with most respondents willing to undergo screening. However, actual uptake was low, with few having previously been screened. Screening methods included visual inspection with acetic acid and Pap smears, and typically occurred once or twice at 3-year intervals. Most believed women of reproductive age should be screened and would recommend it to friends. These results align with Olubodun *et al* [19], Akintobi *et al* [13], Osaro *et al* [15] and a study among female civil servants in Delta state [16], all showing high willingness, while the studies that further assessed the uptake level found low uptake [15, 16]. However, Toye *et al* [21] reported higher uptake, possibly due to targeted interventions in specific subgroups. This discrepancy might be due to targeted education, policies and interventions that might have been in operational among the specific subgroups.

The primary barrier to cervical cancer screening identified in this study was a lack of awareness, with over half of the respondents expressing fear of harm from inadequate equipment or untrained practitioners. Some participants believed they were immune to cervical cancer, while others feared exposure to diseases or found the procedure to be painful, costly or embarrassing. Key facilitators for screening uptake included increased awareness, perceived necessity, integration with general health checks and availability of affordable or free services. While screening services such as Pap smear cost approximately 3,865.00–7,730.00 in Nigerian Naira [24] (2.5-5USD; 12 March, 4:20 UTC. Disclaimer), there exists free screening services in both government and private owned facilities via funding from the government and various non-governmental organisations and institutions. In the study area, the hospital management board had organised a free cervical cancer screening capturing a minimum of 287 women at the state secondary health facility [25]. Furthermore, in a commendable initiative marking World Cancer Day 2023, Ibadan Electricity Distribution Company extended its support beyond electricity provision by offering complimentary cervical cancer screening to 20 women in Osun State [26]. Moreover, Fountain and Cambridge Universities have begun the screening of more than 600 women in the region [27].

These findings reflect significant cultural, social and infrastructural barriers that must be addressed to improve screening rates. Similar barriers were noted by Akintobi *et al* [13] in Ogbomoso, Nigeria, including insufficient health education, lack of knowledge about the screening process, financial constraints and limited access to services. Abotchie and Shokar [18] also highlighted misconceptions about the screening process, such as fear of pain and concerns over loss of virginity. Anyebe *et al* [28] further identified stigma, fear of exposure to male doctors, concerns over potential harm from substandard equipment and the need for spousal approval as barriers to screening among nurses in Zaria.

Furthermore, inferential statistical analyses revealed significant associations between screening history and demographic factors such as age, ethnicity and marital status, as well as knowledge level and perceived susceptibility. Older age groups, Igbos and widowed individuals demonstrated higher screening rates, as did those with adequate knowledge and higher perceived susceptibility. Knowledge has been linked to cervical cancer screening in previous studies from southwestern Nigeria, emphasising the need for targeted educational interventions [13]. Higher perceived susceptibility to cervical cancer has also been associated with screening practices, as noted by Uchendu *et al* [12]. This aligns with theoretical frameworks like the Health Belief Model, which underscores perceived susceptibility’s role in shaping health behaviours [29]. While prior studies [12, 30] have shown formal education correlates with health behaviours, in this study, high educational attainment did not significantly predict screening uptake, likely due to barriers identified. However, the high educational level suggests progress in valuing female education, contrasting with traditional African norms prioritising marriage over education [31].

## Conclusion

The study reveals a gap between cervical cancer screening knowledge and action among mothers in Osogbo, Osun State. Comprehensive awareness campaigns, affordable screening services and integration into maternal health services are recommended. Future research should explore women’s lived experiences through qualitative approaches to better understand factors influencing screening decisions.

## Strengths and limitations

The study findings are vital for crafting targeted awareness campaigns. The findings filling the knowledge gaps in this resource setting while also indicating a positive attitude towards screening can be leveraged by public health initiatives to control the identified barriers, thus, promoting uptake. However, the reliance on self-reported data introduces the potential for inaccuracies, as the mothers may overestimate their knowledge or willingness to participate in screening, affecting the reliability of the findings. Furthermore, the geographic limitation of the study, focusing solely on Osogbo, restricts the generalisability of the findings to a broader population of Osun State or other regions in Nigeria or beyond, where levels of awareness and acceptance might differ.

## Conflicts of interest

The authors have no funding or conflicts of interest to disclose.

## Figures and Tables

**Table 1.** Socio-demographic characteristics of respondents.

Variables	*n* (%)	Cervical cancer screening history		χ^2^	*p*-value
		Yes *n* (%)	No *n* (%)		
Age					
°18–27	99 (32.5)	24 (24.2)	75 (75.8)	13.214	0.004*
°28–37	141 (46.2)	29 (20.6)	112 (79.4)		
°38–47	48 (15.7)	16 (33.3)	32 (66.7)		
°48 and above	17 (5.6)	10 (58.8)	7 (41.2)		
Religion					
°Christianity	193 (63.3)	47 (24.4)	146 (75.6)	0.657	0.72
°Islam	105 (34.4)	30 (28.6)	75 (71.4)		
°Traditional	7 (2.3)	2 (28.6)	5 (71.4)		
Ethnicity					
°Hausa	4 (1.3)	0 (0)	4 (100)	9.736	0.021*
°Igbo	16 (5.2)	8 (50)	8 (50)		
°Yoruba	275 (90.2)	71 (25.8)	204 (74.2)		
°Others	10 (3.3)	0 (0)	10 (100)		
Marital status					
°Single	38 (12.4)	4 (10.5)	34 (89.5)	12.718	0.005*
°Married	254 (83.3)	69 (27.2)	185 (72.8)		
°Divorced	6 (2.0)	1 (16.7)	5 (83.3)		
°Widowed	7 (2.3)	5 (71.4)	2 (28.6)		
Educational level					
°No formal	7 (2.3)	2 (28.6)	5 (71.4)	0.54	0.91
°Primary	10 (3.3)	2 (20)	8 (80)		
°Secondary	55 (18)	16 (29.1)	39 (70.9)		
°Tertiary	233 (76.4)	59 (25.3)	174 (74.7)		
Occupation					
°Student	49 (16.1)	10 (20.4)	39 (79.6)	1.032	0.597
°Employed	193 (63.3)	51 (26.4)	142 (73.6)		
°Unemployed	63 (20.6)	18 (28.6)	45 (71.4)		
Knowledge category					
°Inadequate	162 (46.9)	34 (43)	128 (56.6)	4.347	0.049^f^*
°Adequate	143 (53.1)	45 (57)	98 (43.4)		
Susceptibility					
°Low	170 (55.7)	134 (59.3)	36 (45.6)	3.929	0.036^f^*
°High	135 (44.3)	92 (40.7)	43 (54.4)		

χ^2^: Chi-squared test; *f*: fishers’ exact test;

* :*p* < 0.05

**Table 2.** Respondents’ knowledge about cervical cancer and its screening.

Variables	Yes *n* (%)	No *n* (%)
Symptoms of cervical cancer		
°Vaginal bleeding	169 (55.4)	136 (44.6)
°Vaginal foul-smelling discharge	165 (54.1)	140 (45.9)
°Pain during sex	142 (46.6)	163 (53.4)
°Average response - symptoms	159 (52.0)	146 (48.0)
Risk factors of cervical cancer		
°Acquiring HPV	128 (42.0)	177 (58.0)
°Multiple sex partners	135 (44.3)	170 (55.7)
°Multi parity	100 (32.8)	205 (67.2)
°Early sexual intercourse	112 (36.7)	193 (63.3)
°Long-term oral contraceptive use (OCP)	110 (36.1)	195 (63.9)
°Cigarette smoking	88 (28.9)	217 (71.1)
°Average response - risk factors	112 (36.8)	193 (63.2)
Prevention of cervical cancer		
°Vaccination for HPV	165 (54.1)	140 (45.9)
°Avoidance of multiple sexual partners	206 (67.5)	99 (32.5)
°Avoidance of early sexual intercourse	197 (64.6)	108 (35.4)
°Child spacing	174 (57.0)	131 (43.0)
°Avoidance of long-term contraceptive use	188 (61.6)	117 (38.4)
°Early screening	192 (63.0)	113 (37.0)
°No smoking	195 (63.9)	110 (36.1)
°Average response - prevention	188 (61.7)	117 (38.3)
Treatment of cervical cancer		
°Surgery	158 (51.8)	147 (48.2)
°Chemotherapy	154 (50.5)	151 (49.5)
°Radiotherapy	123 (40.3)	182 (59.7)
°Average response - treatment	145 (47.5)	160 (52.5)
Interval of cervical cancer screening		
°Every year	127 (41.6)	178 (58.4)
°Every 3 years	123 (40.3)	182 (59.7)
°Every 5 years	74 (24.3)	231 (75.7)
°Average response - screening interval	125 (41.0)	
Eligibility of cervical cancer screening		
°Women aged 30 and above	129 (42.3)	176 (57.7)
°Prostitutes	107 (35.1)	198 (64.9)
°Elderly women	100 (32.8)	205 (67.2)
°Average response - eligibility	112 (36.7)	193 (63.3)

**Table 3.** Respondents’ acceptability of cervical cancer screening.

Variables	*n* (%)
Would want to do a pap smear/cervical cancer screening	
°Yes	188 (61.6)
°No	117 (38.4)
Gone for a cervical cancer screening test before	
°Yes	79 (25.9)
°No	226 (74.1)
Number of times screened (*n* = 79)	
°1	63 (79.7)
°2	9 (11.4)
°3	4 (5.1)
°4	3 (3.8)
Interval of screening underwent (*n* = 79)	
°Yearly	16 (20.3)
°Once in 3 years	50 (63.3)
°At random	13 (16.5)
Type of screening underwent (*n* = 79)	
°Direct visual	30 (38.0)
°Inspection	22 (27.8)
°Pap smear	27 (34.2)
I think women of reproductive age would go for cervical cancer screening	
°Yes	216 (70.8)
°No	89 (29.2)
Would recommend cervical cancer screening for a female friend	
°Yes	226 (74.1)
°No	79 (25.9)

**Table 4.** Respondents’ reasons for non-utilisation of cervical cancer screening services (n = 226).

Reasons	Strongly agree *n* (%)	Agree *n* (%)	Total *n* (%)	Strongly disagree *n* (%)	Disagree *n* (%)	Total *n* (%)	Don't know *n* (%)
Fear of harm from poor equipment/experts	61 (27.0)	76 (33.6)	137 (60.6)	19 (8.4)	24 (10.6)	43 (19.0)	46 (20.4)
Lack of awareness of services	58 (25.7)	83 (36.7)	141 (62.4)	17 (7.5)	29 (12.8)	46 (20.4)	39 (17.3)
It is painful	28 (12.4)	57 (25.2)	85 (37.6)	12 (5.3)	29 (12.8)	41 (18.1)	100 (44.2)
Too expensive	25 (11.1)	50 (22.1)	75 (33.2)	20 (8.8)	41 (18.1)	61 (27.0)	90 (39.8)
It is embarrassing	35 (15.5)	32 (14.2)	67 (29.6)	29 (13.0)	61 (27.0)	90 (39.8)	69 (30.5)
I believe I can never have cervical cancer	74 (32.7)	58 (25.7)	132 (58.4)	17 (7.5)	35 (15.5)	52 (23.0)	42 (18.6)
Exposure to other diseases	57 (25.2)	52 (23.0)	109 (48.2)	25 (11.0)	44 (19.5)	69 (30.5)	48 (21.2)
